# Multisystem inflammatory syndrome in children (MIS-C) associated with COVID-19, clinical characteristics: A multi-center observational study from Jordan

**DOI:** 10.1016/j.gloepi.2025.100185

**Published:** 2025-01-17

**Authors:** Marwan Shalabi, Salam Ghanem, Iyad Al-Ammouri, Amirah Daher, Enas Al-zayadneh, Alaa Alsmadi, Mais Ayyoub, Samah Abughanam, Mariam Jabr, Montaha Al-Iede

**Affiliations:** a Department of Pediatrics, School of Medicine, The Hashemite University, Amman, Jordan; b Department of Health, Jordan Field Office, United Nations Relief and Works Agency for Palestine Refugees in the Near East, (UNRWA), Amman, Jordan; c Department of Pediatrics, School of Medicine, The University of Jordan, Amman, Jordan; d Department of Pediatrics and Neonatology, Islamic Hospital, Specialty Hospital, Jordan Hospital, Amman, Jordan

**Keywords:** Pediatric, COVID-19, Critical care, Multisystem inflammatory syndrome temporally associated with COVID-19, SARS-CoV-2, Shock, Toxic shock syndrome

## Abstract

**Objective:**

Multisystem inflammatory syndrome of childhood (MIS-C) is a newly recognized entity associated with COVID-19 in children. The objective was to describe the clinical course for 74 patients diagnosed with this disease.

**Methods:**

A multicenter retrospective study including 5 major hospitals in Jordan was conducted. Data from children admitted with confirmed SARS-CoV-2 infection or were in close contact with confirmed cases were collected. Total of 74 patients were diagnosed with MIS-C. Clinical, laboratory, radiological and therapeutic data were collected by retrospective chart review.

**Results:**

Fever, abdominal pain, hypoxia and other manifestation occurred. Cardiac findings were less common and did not include coronary findings. Treatments were mainly Corticosteroids and IVIG. No mortality was found in this series but serious disease occurred and some patients were admitted to Pediatric Intensive Care Unit.

**Conclusions:**

This study described the epidemiology, clinical course, management, and outcome of MIS-C cases in Jordan. The findings were consistent with what has been described from other regions globally. There was a wide spectrum in the severity of presentation. Abdominal pain was more prevalent and some children were misdiagnosed as surgical acute abdomen.

## Introduction

In December 2019, Wuhan city gained global attention as a center for an outbreak of unexplained pneumonia. A Novel coronavirus was isolated by Jan 7th^,^ 2020, and was recognized as the causative agent for this pneumonia. This virus was later named severe acute respiratory syndrome coronavirus 2 (SARS-CoV-2)[1,2]. The disease was designated later on by World Health Organization(WHO) as coronavirus infectious disease 2019 (COVID-19) in February 2020 [3].

The disease left a significant impact on adult populations worldwide. As of the end of August 2022, about 600 million people were confirmed to have the virus and about 6.5 million succumbed to the disease [3,4]. The disease onset in Jordan lagged many countries due to strict closure policies [5].

The disease, though, has been generally mild in children worldwide including Jordan [6,7]. Many children developed a newly described syndrome a sequela of COVID-19 which overlaps with Kawasaki disease (KD) and Toxic Shock Syndrome (TSS). It was named Multisystem Inflammatory Syndrome in Children (MIS-C). It usually develops about 4–5 weeks following infection with SARS-CoV-2 [[8], [9], [10], [11], [12], [13], [14]].

Although rare, MIS-C can be serious, resulting in multisystem involvement with variable degrees of severity, from a mild, self-limited systemic inflammation to severe symptoms of heart failure, shock, and even death in extremely rare situations.

Pediatric COVID-19 has been described in Jordan [6]. It was observed by the end of 2020 and this would be one of the few case series describing the clinical characteristics of MIS-C in Jordan. There are multiple studies though worldwide describing MIS-C [[15], [16], [17], [18], [19], [20]].

Diagnosing MIS-C can be challenging and remains a diagnosis of exclusion, hence it is often overdiagnosed. Resulting in unwarranted treatments such as anticoagulants, steroids, and IVIG administration. It is an interesting new syndrome and the information about it still evolving.

None of our cases occurred in a neonate and this disease is unlikely to occur in newborns as it was described by other series [16,17]. There is though more literature describing Multisystem inflammatory syndrome in neonates (MIS-N) [[21], [22], [23], [24], [25]].

There is another similar entity that occurs in adults and it is called Multisystem Inflammatory Syndrome in Adults (MIS-A). Cardiac and gastrointestinal findings were more common in the adult variant and it is potentially fatal were deaths reported. It is extremely rare though and only a few cases were reported [26].

## Methodology

### Study design and setting

This is a multicenter, retrospective, observational study conducted at five hospitals in Amman, Jordan. The study included children aged 17 years or younger who were diagnosed with MIS-C from January 1st to January 31st, 2022. The study was approved by the institutional review boards at the respective institutions.

### Data collection

Medical records were reviewed by pediatricians and/or pediatric residents at each participating institution. Data was entered into an Excel sheet at each institution and then combined. Patients were coded, and identifier data were hidden to ensure data confidentiality. Data recorded included demographic data including age at diagnosis, nationality, and gender. This study did not participate in performing any of the laboratory or radiological testing but it was merely a retrospective chart review study of clinical data. Symptoms reviewed including the presence of comorbid cardio-respiratory conditions, allergies, or other chronic medical problems as well as the presenting symptoms of fever, weight loss, cough, dyspnea, cyanosis, rhinorrhea, sore throat, abdominal pain, vomiting, diarrhea, skin rash, myalgia, and others. Laboratory data included blood cell count, C-reactive protein level, liver function parameters, ferritin level, serum creatinine level, results of RT-PCR for SARS-CoV-2 (Zybio molaccu covid-19 detection kit or Core tests Covid-19 Ag Test), and other respiratory viruses from nasopharyngeal aspirate as well as results of IgM/IgG antibodies testing against SARS-CoV-2. Results of other studies included chest radiography, electrocardiography, and echocardiography results. Finally, outcome data included length of hospitalization, length of pediatric intensive care (PICU) stay, days of oxygen requirements, days of ventilator support, use of corticosteroids and/or intravenous immunoglobulins (IVIG), antibiotics, anticoagulants, as well as mortality.

For analysis, the patients were divided into three age groups, below 6 years, 6–13 years, and older than 13 years.

### Viral detection

#### Sample collected

Nasopharyngeal and oropharyngeal swabs were used. Technicians engaged in collecting specimens of SARS-CoV-2 received biosafety training. Personal protective equipment (PPE): N95 masks, goggles, protective clothing, and latex gloves were used. For the oropharyngeal swab, a plastic -rod swab with polypropylene fiber tip to swab the bilateral pharyngeal tonsils and posterior pharyngeal wall. The swab’ tip was dipped into a tube containing 3 ml specimen solution, and the tube was tightened and sent to the laboratory. For nasopharyngeal swabs, the same plastic rod swab was used and inserted into the nasopalatine in the nasal canal, slowly rotated, and taken out. The tip was dipped in a tube containing 3 ml solution as well, the tube was then closed tightly and sent to the laboratory. A nucleic acid extraction kit (Magnetic Bead Method) was used to make extraction and preparation of SARS-CoV-2 RNA of a sample volume of 200 μL. Nucleic acid extraction was simultaneously conducted in negative and positive control.

#### Nucleic acid extraction

Zybio molaccu COVID-19 detection test was used. This test qualitatively detects SARS-CoV-2 RNA in the specimen through one-step Real-Time RT-PCR method. The specimen detection process consists of two steps; 1- SARS-CoV-2 RNA isolation and preparation which can be conducted manually or in semi- or full-automatic instruments, 2- Reverse transcription and PCR amplification. The SARS-CoV-2 RNA prepared in the first step is reverse-transcribed to generate complementary DNA in the RT-PCR reaction system. Qualitative detection of SARS-CoV-2 is realized by monitoring the change of fluorescence signal intensity during RT-PCR amplification.

### Case definition

The case definitions for MIS-C were adopted by WHO [27], the Center for Disease Control and Prevention(CDC) [28], or the Royal College of Pediatrics and Child Health (RCPCH) [29]. In this study we implemented the CDC criteria for case definition.

**Table:** 

CDC case definition for MIS-C [28] • An individual aged <21 years presenting with fever*, laboratory evidence of inflammation**, and evidence of clinically severe illness requiring hospitalization, with multisystem (≥2) organ involvement (cardiac, renal, respiratory, hematologic, gastrointestinal, dermatologic or neurological); AND • No alternative plausible diagnoses; AND • Positive for current or recent SARS-CoV-2 infection by RT-PCR, serology, or antigen test; or exposure to a suspected or confirmed COVID-19 case within the 4 weeks prior to the onset of symptoms. *Fever ≥38.0 °C for ≥24 h, or report of subjective fever lasting ≥24 h **Including, but not limited to, one or more of the following: an elevated C-reactive protein (CRP), erythrocyte sedimentation rate (ESR), fibrinogen, procalcitonin, d-dimer, ferritin, lactic acid dehydrogenase (LDH), or interleukin 6 (IL-6), elevated neutrophils, reduced lymphocytes and low albumin Additional comments: • Some individuals may fulfill full or partial criteria for Kawasaki disease but should be reported if they meet the case definition for MIS-C. • Consider MIS-C in any pediatric death with evidence of SARS-CoV-2 infection.

All patient included in this study fulfilled the CDC criteria for diagnosis of the disease. The diagnosis and treatment for this disease could overlap with Kawasaki disease shock syndrome (KDSS), Kawasaki disease (KD), or toxic shock syndrome (TSS). This disease has a widespread spectrum of clinical and laboratory presentations, so disease diagnosis is not always easy to make.

### Statistical analysis

Analysis was done using IBM SPSS statistics version 25, after aggregation of anonymous data using Excel Microsoft sheets. For Reference to age group norm for laboratory results, we used the Nelson textbook of pediatrics, edition 18. To test for the significance of associations in our analysis we used the “one-way ANOVA test at a 95 % Confidence interval (table-2); to examine the significance of association between the laboratory results and the stratified age groups. We used the Chi-square test at a 95 % Confidence interval (Table 1, Table 3, Table 4,) to test for the significance of association between the other variables examined.

**Table 1:** Bio-demographic Information and Symptoms Stratified by Age Groups.

Variable	0- < 6 years (*n* = 27)	6- < 13 years (*n* = 37)	≥ 13 years (*n* = 10)	All Cases (*n* = 74)	P-value
**Male**	13 (17.6 %)	26 (35.1 %)	8 (10.8 %)	47 (63.5 %)	0.098
**Hospitalization** **≥7 days**	16 (21.6 %)	19 (25.7 %)	4 (5.4 %)	39 (52.7 %)	0.566
**Admission to PICU** **≥5 days**	3 (4.1 %)	3 (4.1 %)	1 (1.4 %)	7 (9.5 %)	0.695
**Fever**	23 (31.9 %)	37 (51.4 %)	9 (12.5 %)	69 (95.8 %)	0.063
**Headache**	4 (6.0 %)	17 (25.4 %)	6 (9.0 %)	27 (40.3 %)	0.030*
**Abdominal Pain**	15 (20.8 %)	31 (43.1 %)	4 (5.6 %)	50 (69.4 %)	0.019*
**Palpitations**	3 (4.3 %)	2 (2.9 %)	5 (7.1 %)	10 (14.3 %)	0.001*
**Hemoptysis**	0(0.0)	0(0.0)	1(1.4)	1(1.4)	0.029*
**Yellowish discoloration**	4(6.8)	0(0.0)	0(0.0)	4(6.8)	0.015*
**pleural effusion**	0(0.0)	10(21.3 %)	0(0.0)	10(21.3)	0.009*

## Results

### Biodemographic and general analysis

A total of 74 patients were diagnosed with MIS-C during the study period, with a male predominance of 47 patients (63.5 %). The mean age was 7 years (±4), and the median was 6 years (range of 2 months, to 17 years). Thirty-nine patients (53 %) were in the age group of 6–13 years, while 26 patients (35 %) were less than 6 years, and 10 patients (13.5 %) were older than 13 years old.

Most admissions occurred during December (42 %), followed by November (15 %). The mean hospital stay was 8 days (range of 1–40 days). Twenty patients (27 %) were admitted to the PICU. The mean PICU admit duration was 5 days (± 3.4), a range of 1–13 days. All patients were alive at discharge from the hospital.

### Clinical presentation

Fever was the most common symptom, occurring in 69 patients (95.8 %). The mean duration of fever on presentation was 8 days (±6.3), with a range of 1–30 days. Cough, when present, was characteristically dry in most patients (15/17 patients reported having a cough, had a dry cough). By using the Pearson Chi-Square test at 95 % CI We found the following symptoms significantly associated with age group stratification and significantly associated with certain age groups than others (headache, hemoptysis, jaundice, abdominal pain, palpitations, pleural effusion), Headache was a common feature among all groups with total percentage of (27 cases out of 67 (40.3 %, *P* = 0.030)hemoptysis only one case occurred at the older age group category (≥ 13), jaundice occurred more in the younger age group of fewer than 6 years (4/27, 15. Abdominal pain was more common in patients 6–13 years of age ((83.8 %, *P* = 0.019) Additionally, pleural effusion was more common in patients 6–13 years of age (10/37 patients, 37.0 %), and palpitation was more common in patients older than 13 (5/10 patients 50 %). Details of presenting symptoms and their frequencies are presented in Table 2.

**Table 2:** Age-related Laboratory Work-up.

Laboratory Test	0- < 6 years (Median [IQR])	6- < 13 years (Median [IQR])	≥ 13 years (Median [IQR])	P-value
**CRP (mg/L)**	133.6 [64.2–175.8]	172.8 [98.4–219.3]	145.3 [89.7–170.2]	0.36
**ESR (mm/L)**	62.6 [42.3–85.1]	59.2 [35.1–78.3]	86.2 [50.4–100.7]	0.15
**LDH (unit/L)**	508.4 [317.2–845.1]	327.8 [245.9–469.7]	840.2 [712.3–975.8]	0.00**
**Ferritin (μg/L)**	489.4 [321.7–800.5]	760.3 [530.9–1143.2]	1456.0 [1023.4–1887.8]	0.01**
**Albumin (g/dL)**	3.3 [3.0–3.8]	3.1 [2.7–3.5]	4.1 [3.8–4.5]	0.01**
**AST (U/L)**	206.5 [132.4–342.7]	42.9 [29.8–57.5]	103.4 [78.6–145.2]	0.05

**Table 3:** Imaging Findings at Ward vs. PICU.

Imaging	Ward (Abnormal/Total, %)	PICU (Abnormal/Total, %)	P-value
**Chest X-Ray**	7/28 (25 %)	13/20 (65 %)	0.01
**Abdominal Ultrasound**	15/22 (68 %)	11/11 (100 %)	0.04

**Table 4:** Treatment Modalities at Ward vs. PICU.

Treatment Modality	Ward (*n* = 31, %)	PICU (*n* = 20, %)	P-value
**Steroid (Methylprednisolone)**	28 (90 %)	20 (100 %)	0.16
**IVIG**	8 (26 %)	8 (40 %)	0.29
**Vitamin K**	2 (7 %)	6 (35 %)	0.01**
**Albumin**	2 (7 %)	10 (50 %)	0.00**
**Vasopressors**	0 (0 %)	17 (85 %)	0.00**

### Laboratory results

All of our patients had evidence of a marked inflammatory state (Table 2). The C-reactive protein (CRP) (positive if ≥ 10 mg/L) was high in all patients for all three age categories with a mean result of (133.6, 172.8, and 145.3) respectively for the three age groups. The erythrocyte sedimentation rate (ESR) (positive if ≥ 40 mm/h.) mean was 62.6, 59.2, and 86.2 respectively for the three age groups. Lactate dehydrogenase (LDH), and Ferritin as well were all markedly elevated in all age groups, showing a significant association with the different age groups (*P*-value 0.00, 0.01) respectively. The most elevated mean of the LDH and Ferritin results were for the older age group “most affected group” with means of (840 unit/l, and 1456 micro g/l) respectively.

The hematological results showed that Neutrophilia occurred in both age groups 0- < 6, and 6- < 13 years old, but the third age group ≥ 13 had a normal mean result. The only hematological result that was significantly associated with the different age groups; had a significant difference between age groups was the Basophils (*P*-value 0.02) as shown in Table 2. The Hepatic and Renal laboratory indicators showed that only three laboratory tests (Creatinine, Albumin, and AST) were significantly associated with different age groups, but the most obvious elevation that was seen among all age groups was in the AST results with the mean result (206.5, 42.9, 103.4 Unit/L) for the three age groups respectively. The age group 0- < 6 years was most affected by the hyponatremia with a mean result of 131.7 mmol/l. The Coagulation Indicators (D-Dimer, Fibrinogen, PT, INR) all showed a dramatic elevation in all age groups for this reason no significant association with age groups was noticed, but worth mentioning that the youngest age group 0- < 6 years was the most affected with the coagulation markers.

### Imaging results

By describing the imaging analysis results (Table-3), we noticed that the only imaging modalities with significant association with the admission level of care “Ward, PICU ”; were the chest x-ray and abdominal ultrasound; *P*-value at 95 % CI (0.01, 0.04) respectively. The chest x-ray showed a wide range of abnormalities (hyperinflation, hilar infiltration, bronchial infiltration, peripheral infiltration, cardiomegaly, pleural effusion, pulmonary edema, lower lobes consolidation, bronchopneumonia), 25 % of the patients who were admitted to the ward had an abnormal chest x-ray finding; while those who were admitted to the PICU had 65 % abnormal x-ray findings. The abdominal ultrasound abnormalities were (ascites, hepatosplenomegaly, mesenteric lymphadenitis, free fluid collection, and signs of appendicitis), 68 % of the patients admitted to the ward had abnormal results; while all of the patients who were admitted to the PICU had one or more of the abnormal findings by the abdominal ultrasound imaging. It is noticed that 82 % and 80 % of patients admitted to the PICU had abnormal results in the abdominal and high-resolution chest CT scans, respectively.

### Treatment modalities

Details of medications used in both PICU and pediatric ward patients are summarized in Table 4. The most used for the treatment of patients with MIS-C is methylprednisolone, which was used in 90 % of patients who were admitted to the pediatric ward and in 100 % of patients admitted to the PICU. IVIG was used in 26 % and 40 %, respectively. The majority of patients received the standard dose of Methylprednisolone of 2 mg/kg/day. The dose of reported IVIG, on the other hand, was 2 g/kg as one dose. Intravenous antibiotics were used by most patients in both wards and PICU (90 % and 95 %, respectively). Antiplatelet dose of aspirin was used in almost half of the patients. As expected, vasopressors were used exclusively for patients who were admitted to the PICU, similarly, albumen infusion was more commonly used in PICU patients. Three-quarters of patients who were admitted to the PICU required oxygen supplementation.

Of note, there is a significant percentage of patients who underwent appendectomy in our series due to suspected appendicitis on presentation with abdominal pain and fever. This was also more common in patients who were admitted to the PICU.

### Comparison between PICU / ward admissions (level of care analysis results indicating the severity of MIS-C)

Our analysis of the severity of MIS-C evident by the level of care, PICU vs. ward admission, was conducted. All the factors had a significant association at a 95 % CI; with the level of care, ward Vs PICU. Factors that were most affected by the level of care are (the age categories, hospitalization duration, fever duration, shortness of breath, hypoxemia, altered mental status, ascites, Shock, pleural effusion, increased troponin, Hyperglycemia, hyponatremia, and acidosis.

The age group most affected by the severity of MIS-C was the 6- < 13-year-old This was reflected in the percentage of admissions to the PICU as it was the highest compared to other age groups who were admitted to the PICU. Specifically speaking, 60 % of the patients who were admitted to the PICU were from the age group 6- < 13 years old. We studied the duration of hospitalization as an indicator reflecting the severity and/or complications. We found that 54.9 % of the total patients had a prolonged hospitalization of more than 7 days. On the other hand, 80 % of the prolonged hospitalized patients were previously admitted to the PICU. By comparing the fever duration, we noticed that 57.1 % of the total patients had a fever duration of less than 8 days, compared to 42.9 % of the patients who had a prolonged course of fever of more than 8 days. While studying other clinical signs by comparing them to the level of care we found that (shortness of breath, bluish discoloration, altered mental status, ascites, shock, and pleural effusion) were the factors significantly associated with the level of care that the site of admission in each hospital “WARD/PICU” at a 95 % CI and all had a less than 0.05 *P*-value.

The most significantly affected factors by the level of care which had a significant difference and a P-value of less than 0.05 at a 95 % CI the following: high Troponin, Hypoglycemia, Hyponatremia, and Hypoxia.

## Discussion

In this study, we described 74 children under the age of 17 years living in Amman, a middle-income city in Jordan, who all met the CDC criteria for COVID-19-related MIS-C. No mortality was reported but 27 % required admission to a PICU.

Most of our cohort were school-age children. Similarly, Feldstein et al., Acevedo et al., and Payne et al. reported the same finding [[30], [31], [32]]. In their review, Kundu et al. found the median age of children with MIS-C to be nine years [33].

We noticed a doubling in the number of patients admitted with MIS-C from November to December 2021, 20 % compared to 40 %. This was explained by the increased number of infected patients with the Omicron (B.1.1.529) variant in Jordan. The first case of the OMICRON variant in Jordan was diagnosed on 10th December 2021 [34]. This was followed by a rapid rise in the weekly number of confirmed COVID-19 patients and reached a peak of 26,000 cases in the third week of January [35]. On 26 November 2021, WHO designated the Omicron (B.1.1.529) variant as a variant of concern based on the evidence that this variant has many mutations that might impact the virus spread and the severity of the illness caused by the virus [36]. An increase in the number of MIS-C cases was reported in the world following the emergence of the Omicron variant, from less than 2 % during the early pandemic to 25 % during the period 27th January to 3rd February 2022 [34]. The majority of the cases were in unvaccinated children [35].

Patients with MIS-C in this study were similar in their characteristics to patients previously reported. Most of our patients with MIS-C were males among all age groups. This was noticed also by Brizuela et al., who reported a higher percentage of MIS-C among boys than girls [36]. In a systematic review of studies from Europe and the United States, it was found that almost 40–70 % of reported patients with MIS-C were male with a median age at onset of the disease of 7–10 years. Similar findings were found by other studies as well [37,38].

In our study, fever was the most frequent symptom upon presentation to the hospital. It was reported and documented in 95.8 % of patients, and a third of them developed a fever for more than eight days. Spiking and persistent fever has been widely reported in the literature to be the major symptom of MIS-C. Generalized malaise and loss of appetite were the second and the third most frequently reported symptoms. Gastrointestinal (GI) manifestations were reported by a high percentage of patients with MIS-C as well; 73 % presented with vomiting and 69 % complained of abdominal pain. Many studies have reported GI involvement as the most apparent attribute of MIS-C [[39], [40], [41], [42]].

In Europe and the United States, children recently infected with SARS-COV-2 have presented with a Kawasaki disease-like condition. Abnormal ECHO has been reported to be more associated with MIS-C than severe acute COVID-19 [43]. The most reported finding in ECHO among our patients was pericardial effusion and mitral regurgitation. None were reported to have coronary artery problems during the early follow-up period of MIS-C. Similarly, a recent study from the United States has demonstrated that the coronary arteries might not be affected in the early phase of MIS-C, as well as early follow-up showed no coronary artery involvement [44].

Generally, the number of pediatric patients requiring PICU admissions due to severe COVID-19 disease is lower than adults [45]. It is not yet well-known why the disease severity and mortality are lower in children than in adults; one hypothesis relates to the lower number of angiotensin 2 converting enzyme receptors in children's respiratory tract and the presence of cross-reaction to previous coronavirus exposure [46]. In our study, we compared the children admitted to PICU and those admitted to the pediatric wards. A total of 20(27 %) patients required PICU admission. The factors associated with a higher risk of PICU admission were a prolonged admission of>7 days, prolonged fever of more than 8 days, respiratory distress, pleural effusion, and presentation with a shock to the emergency department. Similarly, Kiymet et al., reported that 25.9 % of patients with MIS-C included in their study, were admitted to PICU [47].

To date, there are multiple treatments used for pediatric patients with severe COVID-19 including MIS-C. Studies describe the use of systemic corticosteroids, antivirals, intravenous immunoglobulins, and biological agents [48]. Administration of IVIG has been reported to be more frequent than in children with acute COVID-19 infection [43]. Corticosteroids have been reported to reduce mortality in adults with severe COVID-19. Recently, a study reported a beneficial use of anti-inflammatory medications in children with MIS-C as well [43]. In our study, all patients were admitted to PICU, and the majority of the cohort were given at least one dose of IVIG. Furthermore, those who required intensive care were more hypoxic and had higher troponin levels than those who were admitted to the pediatric wards.

Similar to our findings, some studies have reported lymphopenia and thrombocytopenia, Feldstein et al. have reported higher C-reactive protein levels, higher neutrophil to lymphocyte ratio and lower platelets count among children with MIS-C than those with severe acute COVID-19 infection [43].

## Conclusion

Multisystem inflammatory syndrome of childhood (MIS-C) can present in children with a wide spectrum of symptoms from mild disease to a serious one. Early recognition and intervention are essential to treat it. Abdominal pain is a prominent symptom that could lead to unnecessary surgical procedures. These findings in Jordan were like what was seen in other countries.

## CRediT authorship contribution statement

**Marwan Shalabi:** Writing – review & editing, Writing – original draft, Visualization, Validation, Supervision, Resources, Project administration, Methodology, Data curation, Conceptualization. **Salam Ghanem:** Software, Methodology, Formal analysis, Data curation. **Iyad Al-Ammouri:** Writing – original draft, Data curation, Conceptualization. **Amirah Daher:** Writing – original draft, Formal analysis, Data curation. **Enas Al-zayadneh:** Writing – original draft, Data curation, Conceptualization. **Alaa Alsmadi:** Investigation, Data curation, Conceptualization. **Mais Ayyoub:** Methodology, Investigation, Data curation. **Samah Abughanam:** Methodology, Formal analysis, Data curation. **Mariam Jabr:** Methodology, Investigation, Formal analysis, Data curation. **Montaha Al-Iede:** Writing – review & editing, Writing – original draft, Visualization, Validation, Supervision, Project administration, Formal analysis, Data curation.

## Declaration of competing interest

The authors declare no conflict of interest.

## References

[1] Gorbalenya A.E., Baker S.C., Baric R.S., de Groot R.J., Drosten C., Gulyaeva A.A. (2020). Severe acute respiratory syndrome-related coronavirus: the species and its viruses – a statement of the Coronavirus Study Group [Internet]. bioRxiv.

[2] Phelan A.L., Katz R., Gostin L.O. (2020 Feb 25). The novel coronavirus originating in Wuhan, China: challenges for global health governance. JAMA.

[3] Coronavirus disease (COVID-19) – World Health Organization [Internet] https://www.who.int/emergencies/diseases/novel-coronavirus-2019.

[4] Chan J.W.M., Ng C.K., Chan Y.H., Mok T.Y.W., Lee S., Chu S.Y.Y. (2003 Aug). Short term outcome and risk factors for adverse clinical outcomes in adults with severe acute respiratory syndrome (SARS). Thorax.

[5] Al-Tamimi M., Qaisieh R., Tadros R.E., Shalabi M., Alhasoun A., Kilani M.M. (2020 Dec). Successful flattening of COVID-19 epidemiological curve in Jordan. J Glob Health.

[6] Kilani M.M., Odeh M.M., Shalabi M., Al Qassieh R., Al-Tamimi M. (2021 Feb). Clinical and laboratory characteristics of SARS-CoV2-infected paediatric patients in Jordan: serial RT-PCR testing until discharge. Paediatr Int Child Health.

[7] Pathak E.B., Salemi J.L., Sobers N., Menard J., Hambleton I.R. (2020). COVID-19 in children in the United States: intensive care admissions, estimated total infected, and projected numbers of severe pediatric cases in 2020. J Public Health Manag Pract.

[8] Belot A., Antona D., Renolleau S., Javouhey E., Hentgen V., Angoulvant F. (2020 Jun). SARS-CoV-2-related paediatric inflammatory multisystem syndrome, an epidemiological study, France, 1 March to 17 May 2020. Euro Surveill.

[9] Capone C.A., Subramony A., Sweberg T., Schneider J., Shah S., Rubin L. (2020 Sep). Characteristics, cardiac involvement, and outcomes of multisystem inflammatory syndrome of childhood associated with severe acute respiratory syndrome coronavirus 2 infection. J Pediatr.

[10] CDC (2020). https://www.cdc.gov/coronavirus/2019-ncov/index.html.

[11] Grimaud M., Starck J., Levy M., Marais C., Chareyre J., Khraiche D. (2020 Jun 1). Acute myocarditis and multisystem inflammatory emerging disease following SARS-CoV-2 infection in critically ill children. Ann Intensive Care.

[12] McMurray J.C., May J.W., Cunningham M.W., Jones O.Y. (2020). Multisystem inflammatory syndrome in children (MIS-C), a post-viral myocarditis and systemic vasculitis-a critical review of its pathogenesis and treatment. Front Pediatr.

[13] Shulman S.T. (2020 May;). Pediatric coronavirus disease-2019–associated multisystem inflammatory syndrome. J Pediatric Infect Dis Soc.

[14] Waltuch T., Gill P., Zinns L.E., Whitney R., Tokarski J., Tsung J.W. (2020 Oct). Features of COVID-19 post-infectious cytokine release syndrome in children presenting to the emergency department. Am J Emerg Med.

[15] Ahmed M., Advani S., Moreira A., Zoretic S., Martinez J., Chorath K. (2020 Sep). Multisystem inflammatory syndrome in children: a systematic review. EClinicalMedicine.

[16] Belay E.D., Abrams J., Oster M.E., Giovanni J., Pierce T., Meng L. (2021 Aug 1). Trends in geographic and temporal distribution of US children with multisystem inflammatory syndrome during the COVID-19 pandemic. JAMA Pediatr.

[17] Godfred-Cato S., Bryant B., Leung J., Oster M.E., Conklin L., Abrams J. (2020 Aug 14). COVID-19-associated multisystem inflammatory syndrome in children - United States, march-July 2020. MMWR Morb Mortal Wkly Rep.

[18] Matsuda E.M., dos Santos S.A., Castejon M.J., Ahagon C.M., de Campos I.B., de Brígido L.F.M. (2020 Oct 6). COVID-19 in children: a case report of multisystem inflammatory syndrome (MIS-C) in São Paulo, Brazil. Braz J Infect Dis.

[19] Riphagen S., Gomez X., Gonzalez-Martinez C., Wilkinson N., Theocharis P. (2020 May 23). Hyperinflammatory shock in children during COVID-19 pandemic. Lancet.

[20] Torres J.P., Izquierdo G., Acuña M., Pavez D., Reyes F., Fritis A. (2020 Nov). Multisystem inflammatory syndrome in children (MIS-C): report of the clinical and epidemiological characteristics of cases in Santiago de Chile during the SARS-CoV-2 pandemic. Int J Infect Dis.

[21] Divekar A.A., Patamasucon P., Benjamin J.S. (2021 May). Presumptive neonatal multisystem inflammatory syndrome in children associated with coronavirus disease 2019. Am J Perinatol.

[22] Diwakar K., Gupta B.K., Uddin M.W., Sharma A., Jhajra S. (2022). Multisystem inflammatory syndrome with persistent neutropenia in neonate exposed to SARS-CoV-2 virus: a case report and review of literature. J Neonatal Perinatal Med.

[23] More K., Aiyer S., Goti A., Parikh M., Sheikh S., Patel G. (2022 May). Multisystem inflammatory syndrome in neonates (MIS-N) associated with SARS-CoV2 infection: a case series. Eur J Pediatr.

[24] Pawar R., Gavade V., Patil N., Mali V., Girwalkar A., Tarkasband V. (2021 Jul 2). Neonatal multisystem inflammatory syndrome (MIS-N) associated with prenatal maternal SARS-CoV-2: a case series. Children (Basel).

[25] Diggikar S., Nanjegowda R., Kumar A., Kumar V., Kulkarni S., Venkatagiri P. (2022 May). Neonatal multisystem inflammatory syndrome secondary to SARS-CoV-2 infection. J Paediatr Child Health [Internet].

[26] Vogel T.P., Top K.A., Karatzios C., Hilmers D.C., Tapia L.I., Moceri P. (2021 May 21). Multisystem inflammatory syndrome in children and adults (MIS-C/A): case definition & guidelines for data collection, analysis, and presentation of immunization safety data. Vaccine.

[27] Multisystem inflammatory syndrome in children and adolescents with COVID-19 [Internet]. https://www.who.int/publications-detail-redirect/multisystem-inflammatory-syndrome-in-children-and-adolescents-with-covid-19.

[28] CDC (2020). https://www.cdc.gov/mis/mis-c/hcp/index.html.

[29] Paediatric multisystem inflammatory syndrome temporally associated with COVID-19 (PIMS) - guidance for clinicians [Internet]. RCPCH. [cited 2023 Feb 12]. Available from: https://www.rcpch.ac.uk/resources/paediatric-multisystem-inflammatory-syndrome-temporally-associated-covid-19-pims-guidance.

[30] Acevedo L., Piñeres-Olave B.E., Niño-Serna L.F., Vega L.M., Gomez I.J.A., Chacón S. (2021 Nov 18). Mortality and clinical characteristics of multisystem inflammatory syndrome in children (MIS-C) associated with covid-19 in critically ill patients: an observational multicenter study (MISCO study). BMC Pediatr.

[31] Feldstein L.R., Rose E.B., Horwitz S.M., Collins J.P., Newhams M.M., Son M.B.F. (2020 Jul 23). Multisystem inflammatory syndrome in U.S. children and adolescents. N Engl J Med.

[32] Payne A.B., Gilani Z., Godfred-Cato S., Belay E.D., Feldstein L.R., Patel M.M. (2021 Jun 10). Incidence of multisystem inflammatory syndrome in children among US persons infected with SARS-CoV-2. JAMA Netw Open.

[33] Kundu A., Maji S., Kumar S., Bhattacharya S., Chakraborty P., Sarkar J. (2022). Clinical aspects and presumed etiology of multisystem inflammatory syndrome in children (MIS-C): a review. Clin Epidemiol Glob Health.

[34] (2021). Jordan health ministry detects its first two cases of Omicron COVID-19 variant [Internet].

[35] (2022). 4th wave of COVID to reach peak by mid-February, warns top health official [Internet].

[36] Brizuela M., Lenzi J., Ulloa-Gutiérrez R., Yassef A.M.O., Aida J.A.R., del Aguila O. (2021). Influence of sex on disease severity in children with COVID-19 and Multisystem Inflammatory Syndrome in Latin America [Internet]. medRxiv.

[37] Belay E.D., Godfred-Cato S. (2022 May). SARS-CoV-2 spread and hospitalisations in paediatric patients during the omicron surge. Lancet Child Adolesc Health.

[38] Zhu F., Ang J.Y. (2022). COVID-19 infection in children: diagnosis and management. Curr Infect Dis Rep.

[39] Nakra N.A., Blumberg D.A., Herrera-Guerra A., Lakshminrusimha S. (2020 Jul 1). Multi-system inflammatory syndrome in children (MIS-C) following SARS-CoV-2 infection: review of clinical presentation, hypothetical pathogenesis, and proposed management. Children (Basel).

[40] Radia T., Williams N., Agrawal P., Harman K., Weale J., Cook J. (2021 Jun). Multi-system inflammatory syndrome in children & adolescents (MIS-C): a systematic review of clinical features and presentation. Paediatr Respir Rev.

[41] Reiff D.D., Mannion M.L., Samuy N., Scalici P., Cron R.Q. (2021 Feb 24). Distinguishing active pediatric COVID-19 pneumonia from MIS-C. Pediatr Rheumatol Online J.

[42] Rothan H.A., Byrareddy S.N. (2021 Jan). The potential threat of multisystem inflammatory syndrome in children during the COVID-19 pandemic. Pediatr Allergy Immunol.

[43] Feldstein L.R., Tenforde M.W., Friedman K.G., Newhams M., Rose E.B., Dapul H. (2021 Mar 16). Characteristics and outcomes of US children and adolescents with multisystem inflammatory syndrome in children (MIS-C) compared with severe acute COVID-19. JAMA.

[44] Matsubara D., Kauffman H.L., Wang Y., Calderon-Anyosa R., Nadaraj S., Elias M.D. (2020 Oct 27). Echocardiographic findings in pediatric multisystem inflammatory syndrome associated with COVID-19 in the United States. J Am Coll Cardiol.

[45] Chao J.Y., Derespina K.R., Herold B.C., Goldman D.L., Aldrich M., Weingarten J. (2020 Aug). Clinical characteristics and outcomes of hospitalized and critically ill children and adolescents with coronavirus disease 2019 at a tertiary care medical center in New York City. J Pediatr.

[46] Traiber C., Bueno F.U., Braun Filho L.R., Eckert G.U., Azambuja M.A., Gras G.S. (2022 Jun). Pediatric patients with COVID-19 admitted to a PICU in Southern Brazil, excluding MIS-C. Acta Colomb Cuidado Intens.

[47] Kıymet E., Böncüoğlu E., Şahinkaya Ş., Cem E., Çelebi M.Y., Düzgöl M. (2021 Dec 8). A comparative study of children with MIS-C between admitted to the pediatric intensive care unit and pediatric ward: a one-year retrospective study. J Trop Pediatr.

[48] Kabeerdoss J., Pilania R.K., Karkhele R., Kumar T.S., Danda D., Singh S. (2021 Jan). Severe COVID-19, multisystem inflammatory syndrome in children, and Kawasaki disease: immunological mechanisms, clinical manifestations and management. Rheumatol Int.

