# Corrigendum to <Multisystem Inflammatory Syndrome in Children (MIS-C) Associated with COVID-19, Clinical Characteristics: A Multi-Center Observational Study from Jordan> <[9 (2025), 100185]>

**DOI:** 10.1016/j.gloepi.2025.100215

**Published:** 2025-09-03

**Authors:** Marwan Shalabi, Salam Ghanem, Iyad Al-Ammouri, Amirah Daher, Enas Al-zayadneh, Alaa Alsmadi, Mais Ayyoub, Samah Abughanam, Mariam Jabr, Montaha AL-Iede

**Affiliations:** a Department of Pediatrics, School of Medicine, The Hashemite University, Zarqa, Jordan; b Department of Health, Jordan Filed Office, United Nations Relief and Works Agency for Palestinian Refugees in the Near East, Amman, Jordan; c Department of Pediatrics, School of Medicine, The University of Jordan, Amman, Jordan; d Department of Pediatrics and Neonatology, Islamic Hospital, Specialty Hospital, Jordan Hospital, Amman, Jordan

The authors regret having the change done in the city of the main author's university location. It should be Zarqa, Jordan, not Amman, Jordan.

The authors would like to apologise for any inconvenience caused.

Thank you for understanding.

